# Electroacupuncture is Beneficial for Primary Dysmenorrhea: The Evidence from Meta-Analysis of Randomized Controlled Trials

**DOI:** 10.1155/2017/1791258

**Published:** 2017-12-05

**Authors:** Si-yi Yu, Zheng-tao Lv, Qing Zhang, Sha Yang, Xi Wu, You-ping Hu, Fang Zeng, Fan-rong Liang, Jie Yang

**Affiliations:** ^1^The 3rd Teaching Hospital, Chengdu University of Traditional Chinese Medicine, Chengdu 610075, China; ^2^Department of Orthopedics, Tongji Hospital, Tongji Medical College, Huazhong University of Science and Technology, Wuhan, China

## Abstract

Electroacupuncture (EA) is considered to be a promising alternative therapy to relieve the menstrual pain for primary dysmenorrhea (PD), but the conclusion is controversial. Here, we conducted a systematic review and meta-analysis specifically to evaluate the clinical efficacy from randomized controlled trials (RCTs) on the use of EA in patients with PD. PubMed, Embase, ISI Web of Science, CENTRAL, CNKI, and Wanfang were searched to identify RCTs that evaluated the effectiveness of EA for PD. The outcome measurements included visual analogue scale (VAS), verbal rating scale (VRS), COX retrospective symptom scale (RSS), and the curative rate. Nine RCTs with high risk of bias were included for meta-analysis. The combined VAS 30 minutes after the completion of intervention favoured EA at SP6 when compared with EA at GB39, nonacupoints, and waiting-list groups. EA was superior to pharmacological treatment when the treatment duration lasted for three menstrual cycles, evidenced by significantly higher curative rate. No statistically significant differences between EA at SP6 and control groups were found regarding the VRS, RSS-COX1, and RSS-COX2. The findings of our study suggested that EA can provide considerable immediate analgesia effect for PD. Additional studies with rigorous design and larger sample sizes are needed.

## 1. Introduction

Primary dysmenorrhea (PD), or menstrual pain without discernable organic causes, is the most prevalent gynecologic complaint in young women. The prevalence of primary dysmenorrhea ranges from 20% to 90% of menstruating women [1], with severe pain perceived in 10% to 20% of the studied female adolescents [2]. The painful menstrual cramps experienced by women with PD can be considerably disabling, having been likened to renal colic pain [3]. Previous studies have proposed that PD is associated with prostaglandins (PGs), which may play a vital role in modulating hyperalgesia and inflammatory pain and causing uterine contraction [4]. Although PD is not life-threatening, the degree of pain severely affects women's quality of life and ability to study or work normally [5, 6]. Therefore, an improved understanding and treatment for PD are urgent and compulsory.

Nowadays, the treatment for PD includes a variety of pharmacological and nonpharmacological methods. On account of the PG-based etiology of PD, the current frontline pharmacological treatment for PD is nonsteroidal anti-inflammatory drugs (NSAIDs) [7]. Undeniably, NSAIDs have well-documented efficacy against menstrual pain; however, they have a high failure rate of 20%–30% [8]. Moreover, painkillers are also associated with an increased risk of adverse events (AEs), including the digestive tract, liver, and kidney [9]. Accordingly, the search for a low-risk and effective nonpharmacological therapy to relieve menstrual pain represents urgent clinical demand.

Acupuncture, an integral part of Traditional Chinese Medicine (TCM) [10], has been verified to be effective in relieving the menstrual pain and reducing the symptoms of primary dysmenorrhea through plenty of randomized controlled trials (RCTs) [11–13] and systematic reviews [14, 15]. As one of the oldest nonpharmacological therapies, manual acupuncture (MA) mediates its therapeutic effect through the insertion of needles into specific points in the body called acupoints [16]. In the past decades, electroacupuncture (EA), as a means combining traditional acupuncture with modern electrotherapy, has been widely used in China and elsewhere around the world due to its accurate, quantifiable, and repeatable parameters of intensity, frequency, and duration [17, 18]. So far, the mechanism of EA for PD has not been fully illuminated, but some experimental investigations indicated that the EA could reduce the expression levels of prostaglandin (PGF2*α*) [19], the IP3 [20], and the peripheral blood T lymphocyte subsets [21] in a rat model of PD. To date, although many RCTs have demonstrated significant efficacy and safety of EA for PD [22], further investigations are required to draw a reliable conclusion about the effectiveness of this treatment.

However, no published systematic review focused specifically on the effect of EA for PD. Although the results of several published systematic reviews with meta-analysis [14, 15, 23–27] of acupuncture for PD suggested a positive effect of EA, their results could not be confirmed partially due to the small number and methodological flaws of the included studies [22, 28–30]. Moreover, both manual acupuncture and electrical acupuncture are typically lumped together to constitute scientific evidence on “acupuncture” among all of the above systematic reviews. Significantly, Langevin et al. recently reported that EA and MA treatments are not interchangeable and thus must be separately identified for accurate study [31]. Further, Zhang et al. summarized the mechanisms of MA-EA on persistent pain, indicating that EA displays greater analgesic effects for different types of pain in comparison to MA [32]. Therefore, with the publication of a fair number of studies on EA for PD in recent years, we conducted this systematic review to critically evaluate the current evidence from RCTs on the use of EA in patients with PD.

## 2. Methods

This systematic review was performed according to the Preferred Reporting Items for Systematic Reviews and Meta-Analyses (PRISMA) guidelines.

### 2.1. Literature Search Strategy

Six electronic databases, PubMed, Embase, ISI Web of Science, CENTRAL, CNKI, and Wanfang, were searched to identify potentially eligible studies. All the above databases were searched from inception up to the latest issue (April 2017) without language restriction. Free text terms and Medical Subject Headings (MeSH) were combined for literature retrieval; MeSH terms were modified according to the specification of each database. The literature search strategy was as follows: (“Electroacupuncture” [Mesh] or electroacupuncture or electric acupuncture) and (“Dysmenorrhea” [Mesh] or dysmenorrhea or menstrual pain or painful menstruation). In CNKI and Wanfang, we searched “Dian zhen” and “Tong jing” for potential eligible Chinese publications. The bibliographies of relevant systematic reviews were also manually searched to ensure that all relevant studies could be included.

### 2.2. Types of Participants

To be included in our systematic review and meta-analysis, participants enrolled in included studies should be diagnosed as primary dysmenorrhea. Following conditions leading to secondary dysmenorrhea, such as endometriosis, uterine myoma, ovarian cyst, intrauterine synechia, or intrauterine devices, should be excluded from the study.

### 2.3. Types of Intervention

Patients in experimental groups were required to receive EA treatment; no specific restriction on current intensity was imposed. RCTs that combined EA and other therapeutic approaches to treat women with PD were excluded.

### 2.4. Types of Control

Patients with PD in control groups mainly received pharmacological treatments, EA at unrelated acupoints, or EA at nonacupoint locations; no specific type of analgesics was imposed. Patients in waiting-list groups were also included.

### 2.5. Outcome Measurements

The primary outcome measure was subjective pain measured by a standard 100 mm VAS 30 minutes after the completion of intervention. We selected verbal rating scale (VRS) and COX retrospective symptom scale (RSS) as the secondary outcome. The latter is a menstrual symptom measure with high reliability and sensitivity; it is comprised of two subscales, a total frequency rating score (RSS-COX1) and an average severity score (RSS-COX2). The 7-point VRS defines menstrual pain according to the limitation of ability to daily work, systematic symptoms, and need for additional analgesics. The curative rate was also included in our meta-analysis.

### 2.6. Types of Publication

Only RCTs regarding the efficacy of EA on PD were included in our study. Case control studies, cohort studies review articles, and animal experiments were excluded.

### 2.7. Data Extraction

Two investigators (Si-yi Yu and Zheng-tao Lv) screened each article independently and each was blinded to the findings of the other reviewer. According to the predetermined inclusion criteria, two reviewers performed strict screening to identify qualified articles independently, and they extracted data from these eligible articles using a standardized data collection form, which included first author, year of the publication, sample size in each group, details of EA intervention and control treatment, main outcome assessment, and duration of treatment.

Any disagreement between the two reviewers was resolved through discussion until a consensus was reached. The third review author (Fan-rong Liang) was consulted if a consensus could not be reached.

### 2.8. Risk of Bias Assessment

The Cochrane Collaboration's tool was employed for the assessment of risk of bias in selected RCTs, which was based on seven items: random sequence generation, allocation concealment, blinding of participants and personnel, blinding of outcome assessment, incomplete outcome data, selective reporting, and other sources of bias. Two reviewers assessed the risk of bias among studies independently; the results were compared afterwards. Disagreements regarding the risk of bias assessment were settled by discussion and consensus between reviewers.

### 2.9. Statistical Analysis

The enrolled participants were dichotomized into cured and not cured to express the intervention effect; risk ratio (RR) and the associated 95% confidence intervals (CIs) were calculated for clinical effect. For continuous variables, mean difference (MD) for changes from baseline was calculated using the same methodology. A *P* value less than 0.1 from the *Q* statistic was considered as statistically significant heterogeneity. *I*^2^ values of 0%, 25%, 50%, and 75% corresponded to no, low, moderate, and high levels of heterogeneity, respectively [33]. The consistency of results across studies was assessed by *τ*^2^ statistic for relevant heterogeneity [34] and *I*^2^ statistic for statistical heterogeneity [33]. We pooled the estimates using the fixed-effects model when no significant heterogeneity was detected [35]. Otherwise, a random-effects model was used [36].

Subgroup analysis by control intervention was conducted. Sensitivity analysis was conducted by removing studies with relatively small sample sizes (less than 20 subjects in EA group or control group) to determine whether small sample sizes contributed to the between-study heterogeneity. Forest plots and funnel plots were generated via RevMan 5.3 (Copenhagen: the Nordic Cochrane Centre, the Cochrane Collaboration, 2014).

## 3. Results

### 3.1. Literature Search

**Figure 1:**
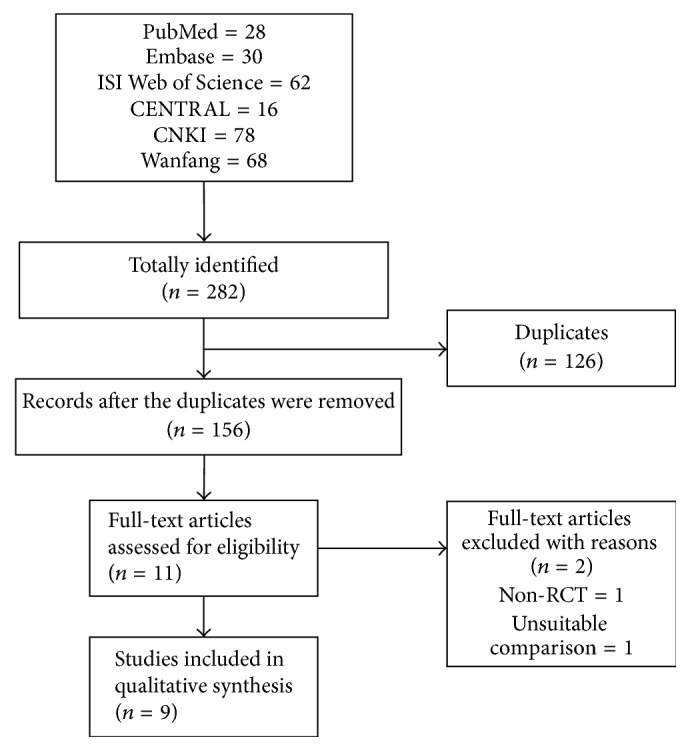
Flow diagram of literature selection.

The systematic literature search yielded a total of 282 potentially relevant articles: 28 from PubMed, 30 from Embase, 62 from ISI Web of Science, 16 from CENTRAL, 78 from CNKI, and 68 from Wanfang. After the removal of duplicates, 156 studies entered the stage of title and abstract screening and 11 of them were downloaded for the full-text screening. Two studies were subsequently removed because one was non-RCT and the other used unsuitable comparison. Additionally, we searched seven systematic reviews related to acupuncture therapy and PD; their included studies that used EA to treat PD were also selected by our current systematic review. Finally, nine studies [28, 30, 37–43] were deemed eligible for inclusion in the meta-analysis. The flow chart of literature search was presented in Figure 1.

### 3.2. Main Characteristics of Included Studies

All the selected RCTs were carried out in mainland China and published from 2007 to 2016. Women with a history of regular menstruation who were diagnosed as PD were enrolled in our included studies. Among these RCTs, SP6 was the most commonly selected acupoint for EA administration. The duration of EA treatment was noted to vary; three two-arm parallel studies [39, 40, 43] compared EA with pharmacological therapy for three menstrual cycles. In these aforementioned studies, EA interventions were introduced from the first day of the onset of menstruation and continued for another four days. After three months' treatment, the clinical effect of EA treatment was evaluated using a dichotomous scale (cured and not cured). The remaining six studies [28, 30, 37, 38, 41, 42] were performed based on three-arm or four-arm parallel design; pain intensity was assessed 30 minutes after the completion of EA administration. The main characteristics of included RCTs were summarized in Table 1.

### 3.3. Methodological Quality

**Figure 2:**
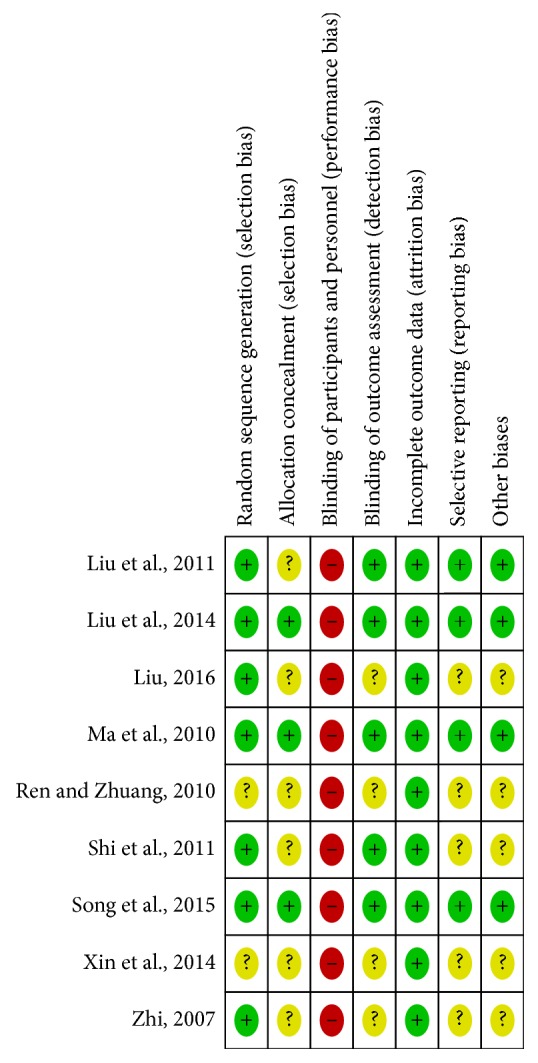
Risk of bias summary: reviewing authors' judgements about each risk of bias item for each included study.

Cochrane's Handbook was utilized for the assessment of methodological quality. All the included studies reported the suggested randomization, but two [40, 42] studies failed to provide the method of random sequence generation. Regarding the allocation concealment, three studies [28, 37, 41] reported the details of allocation concealment procedure. When it comes to blinding of personnel and participants, all trials were judged to have a high risk of bias because it was unfeasible to blind the acupuncturists who administered EA. In terms of blinding of outcome assessment, five studies [28, 30, 37, 38, 41] were judged to have a low risk of bias. The reviewers' judgements about each risk of bias item were presented in Figure 2.

### 3.4. VAS

**Figure 3:**
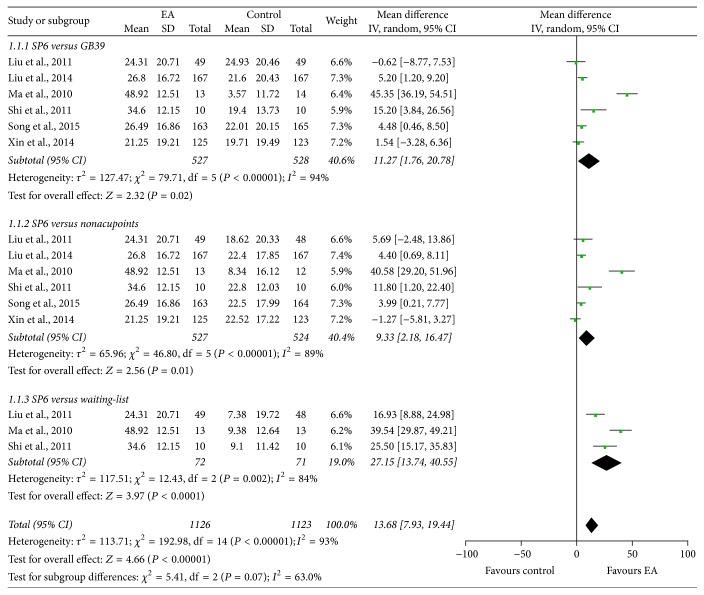
Forest plot of electroacupuncture versus control: VAS.

In order to determine the immediate analgesic effect of EA on PD, six studies [28, 30, 37, 38, 41, 42] evaluated menstrual pain 30 minutes after the end of first EA administration using the 100 mm VAS. Significant heterogeneity among included studies was observed; thus the random-effects model was used. The pooled results showed that EA at SP6 was better in pain relief compared to EA at GB39 (*τ*^2^ = 127.47, *χ*^2^ = 79.71, df = 5, and *I*^2^ = 94%; MD: 11.27; 95% CI: 1.76, 20.78), EA at nonacupoints (*τ*^2^ = 65.96, *χ*^2^ = 46.80, df = 5, and *I*^2^ = 89%; MD: 9.33; 95% CI: 2.18, 16.47), and waiting-list (*τ*^2^ = 117.51, *χ*^2^ = 12.43, df = 2, and *I*^2^ = 84%; MD: 27.15; 95% CI: 13.74, 40.55) groups (Figure 3).

### 3.5. VRS

**Figure 4:**
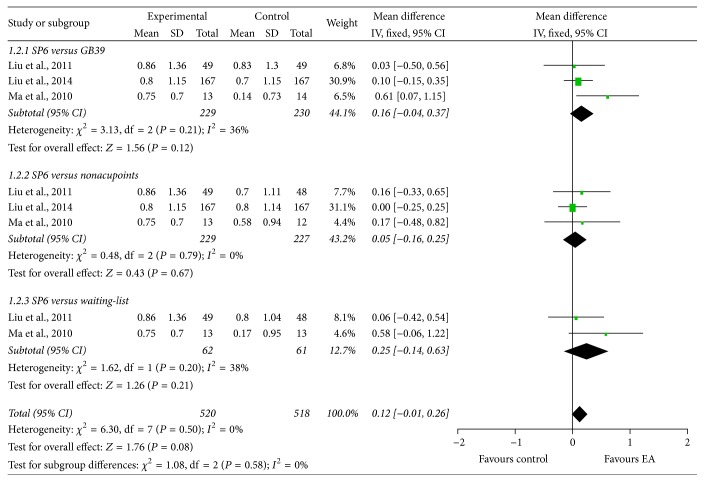
Forest plot of electroacupuncture versus control: VRS.

The 7-point VRS was employed by three studies [28, 30, 37] to assess menstrual pain in the intervention menstrual cycle based on loss of efficiency of work, systematic symptoms, and need for additional analgesics. Compared with GB39 (*χ*^2^ = 3.13, df = 2, and *I*^2^ = 36%; MD: 0.16; 95% CI: −0.04, 0.37), nonacupoints (*χ*^2^ = 0.48, df = 2, and *I*^2^ = 0%; MD: 0.05; 95% CI: −0.16, 0.25), and waiting-list (*χ*^2^ = 1.62, df = 1, and *I*^2^ = 38%; MD: 0.25; 95% CI: −0.14, 0.63) groups, EA at SP6 showed no better effect in improving VRS score (Figure 4).

### 3.6. RSS-COX1 and RSS-COX2

**Figure 5:**
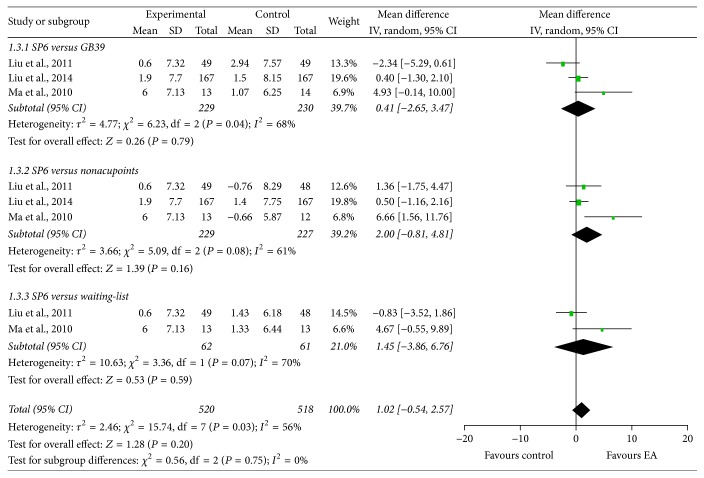
Forest plot of electroacupuncture versus control: RSS-COX1.

RSS-COX1 refers to the total frequency of menstrual pain conditions that patients experienced in the intervention menstrual cycle, with lower score indicating better health. Obvious between-study heterogeneity was detected, so the random-effects model was used. The combined results from three studies [28, 30, 37] suggested that EA at SP6 had equivalent effect to EA at GB39 (*τ*^2^ = 4.77, *χ*^2^ = 6.23, df = 2, and *I*^2^ = 68%; MD: 0.41; 95% CI: −2.65, 3.47), EA at nonacupoints (*τ*^2^ = 3.66, *χ*^2^ = 5.09, df = 2, and *I*^2^ = 61%; MD: 2.00; 95% CI: −0.81, 4.81), and waiting-list (*τ*^2^ = 10.63, *χ*^2^ = 3.36, df = 1, and *I*^2^ = 70%; MD: 1.45; 95% CI: −3.86, 6.76) groups in reducing the total frequency of menstrual pain conditions (Figure 5).

**Figure 6:**
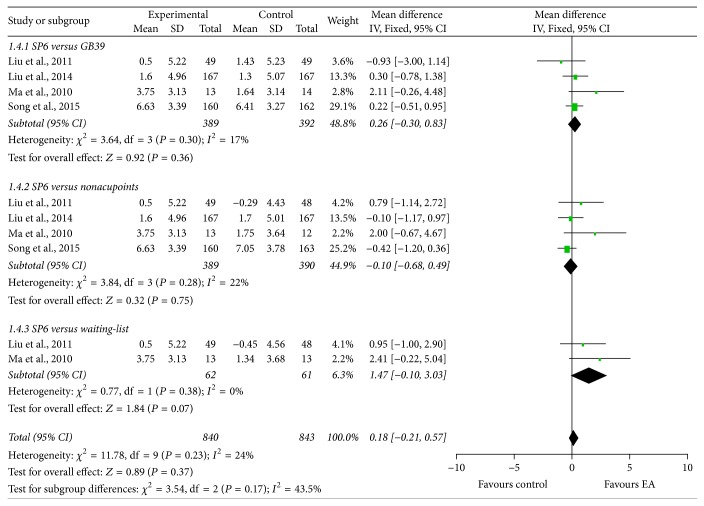
Forest plot of electroacupuncture versus control: RSS-COX2.

Four studies [28, 30, 37, 41] used RSS-COX2 to evaluate the average severity of menstrual pain in the intervention menstrual cycle. Since no significant heterogeneity across studies was observed, the fixed-effects model was employed for statistical analysis. The combined results indicated that EA at SP6 had similar effect in improving RSS-COX2 scores compared with EA at GB39 (*χ*^2^ = 3.64, df = 3, and *I*^2^ = 17%; MD: 0.26; 95% CI: −0.30, 0.83), EA at nonacupoints (*χ*^2^ = 3.84, df = 2, and *I*^2^ = 22%; MD: −0.10; 95% CI: −0.68, 0.49), and waiting-list (*χ*^2^ = 0.77, df = 1, and *I*^2^ = 0%; MD: 1.47; 95% CI: −0.10, 3.03) groups (Figure 6).

### 3.7. Curative Rate

**Figure 7:**
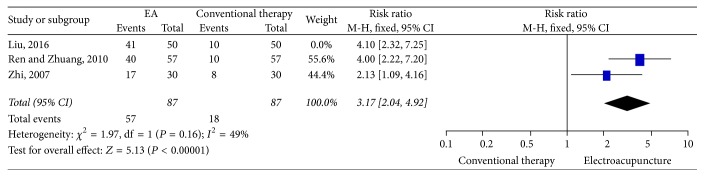
Forest plot of electroacupuncture versus control: curative rate.

Three RCTs [39, 40, 43] adopted curative rate as outcome assessment; all of these three studies compared EA therapy with conventional drug therapy. In the studies of Ren and Zhuang and Zhi, patients in control groups received 600 mg ibuprofen daily, while in Liu's study women received Tianqi Tongjing Capsules to alleviate menstrual pain. After three menstrual cycles' treatment, patients with PD were dichotomized as cured and not cured according to the “Standards for Diagnosis of Syndromes or Diseases of TCM and Evaluation of the Therapeutic Effect” [44]. "Cured" was defined as complete pain relief after three months' treatment without recurrence. "Not cured" referred to the situations where menstrual pain was not relieved and other related symptoms were not alleviated after three months' treatment. The study by Liu et al. was not included in the meta-analysis due to the uncertainty of the clinical effect of Tianqi Tongjing Capsule in PD. Liu and colleagues reported that EA at ST34 and ST36 was significantly better than Tianqi Tongjing Capsule in improving the curative rate (RR: 4.10; 95% CI: 2.32, 9.25). Since no obvious between-study heterogeneity existed (*P* = 0.16; *I*^2^ = 49%), the fixed-effects model was used for meta-analysis. The combination of curative rate suggested that EA was superior to ibuprofen when the treatment duration lasted for three menstrual cycles (*χ*^2^ = 1.97 and df = 1; RR: 3.17; 95% CI: 2.04, 4.92; *P* < 0.00001) (Figure 7).

### 3.8. Sensitivity Analysis

**Figure 8:**
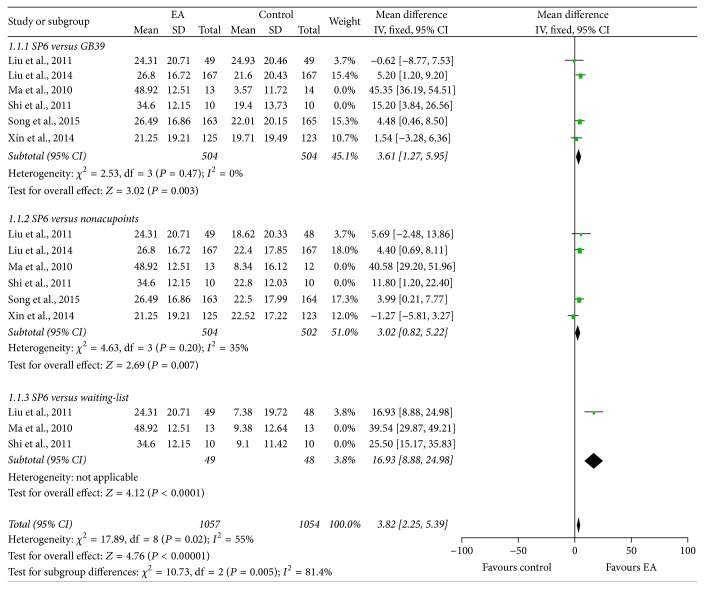
Sensitivity analysis by removing studies with relatively small sample sizes.

Sensitivity analysis was conducted by removing RCTs with relatively small sample size and reevaluating the resulting effect. In the forest plot of VAS score, after the removal of Ma et al. and Shi et al. studies, heterogeneity across studies significantly decreased, and the conclusion regarding the immediate analgesic effect of EA at SP6 did not change (Figure 8).

### 3.9. Publication Bias

**Figure 9:**
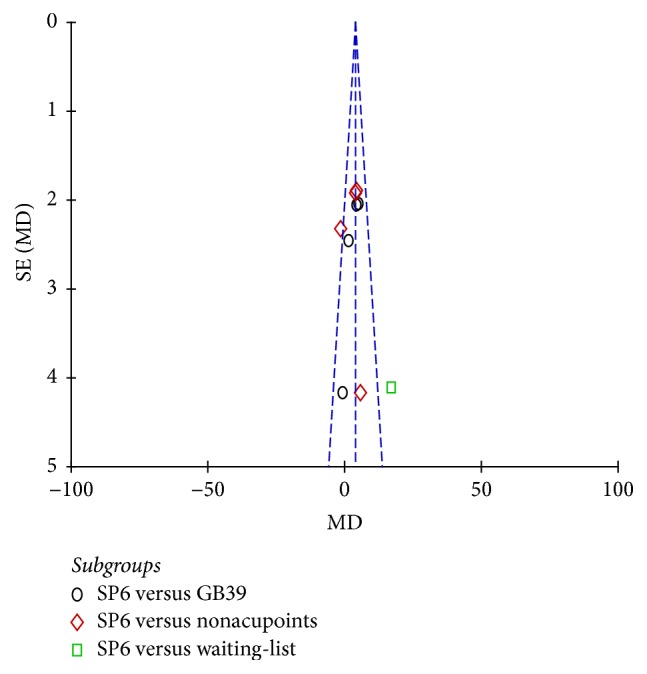
Funnel plot of electroacupuncture versus control: VAS.

**Figure 10:**
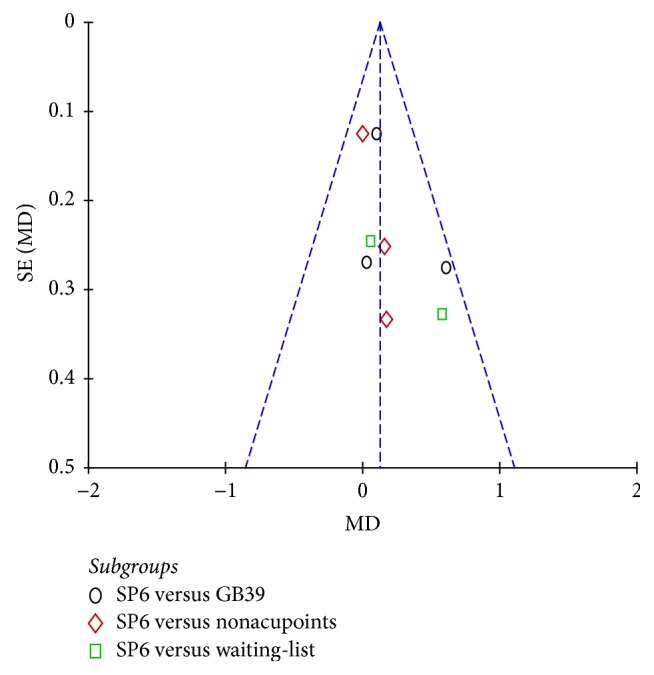
Funnel plot of electroacupuncture versus control: VRS.

**Figure 11:**
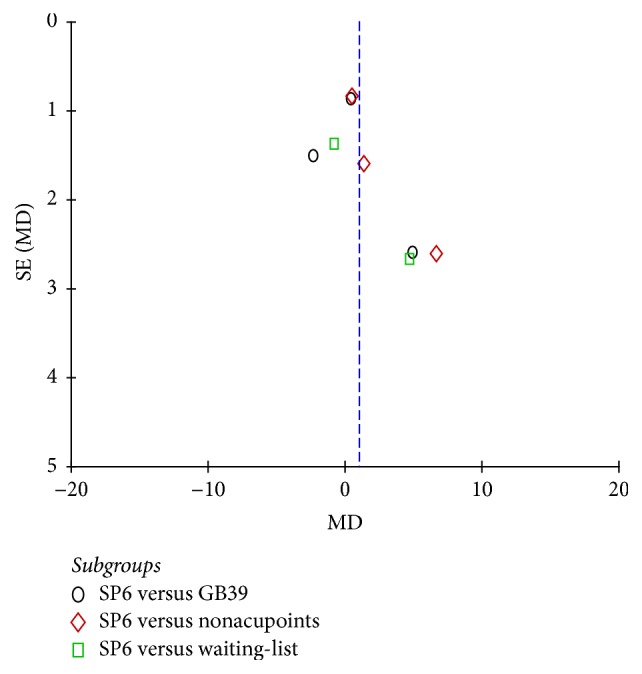
Funnel plot of electroacupuncture versus control: RSS-COX1.

**Figure 12:**
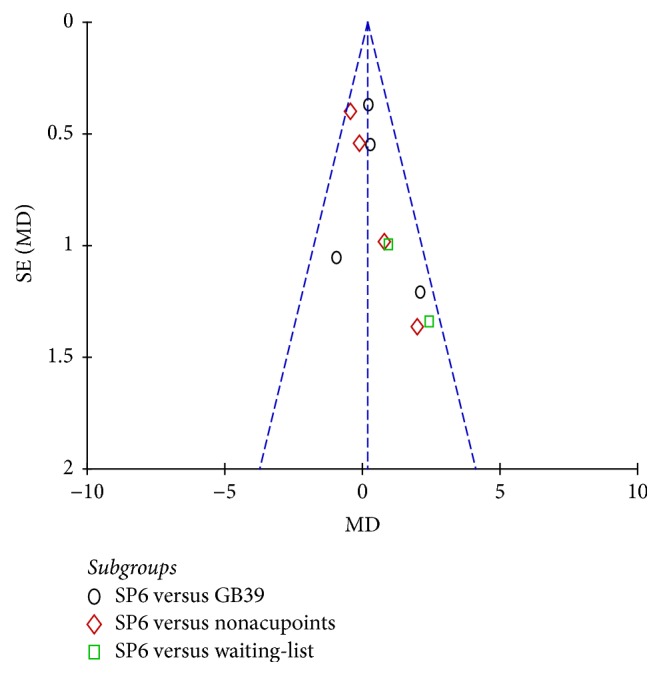
Funnel plot of electroacupuncture versus control: RSS-COX2.

The funnel plots of VAS, VRS, RSS-COX1, and RSS-COX2 were presented in Figures 9, 10, 11, and 12. All the funnel plots presented no asymmetry, suggesting no obvious publication bias.

## 4. Discussion

### 4.1. Overview of Findings

A total of nine studies involving 1951 participants examining the effects of EA therapy on the management of PD were identified in this systematic review and meta-analysis. In our meta-analysis, the immediate and long-term therapeutic effects of EA were evaluated through an analysis of six and three pooled RCTs, respectively. In terms of pain intensity, six studies reported positive results using the VAS [28, 30, 37, 38, 41, 42], suggesting that EA at SP6 acupoint had a significant immediate effect on menstrual pain compared with treatment-irrelevant acupoint (GB39), nonacupoint, and waiting-list control. The goal of therapy is to minimize the pelvic pain that starts with the onset of the menstrual flow. Currently, our results suggest that EA stimulation at classic acupoint could alleviate the pain at once when compared with controls. The immediate analgesic effects of EA may be associated with the activation of the endogenous opioid system, which has been supported by plenty of experimental evidence [45–47].

*Sanyinjiao *(SP6), located medially four-finger wide above the ankle, has been the major acupoint to be used for treating PD since ancient times according to the meridian theory of Chinese acupuncture. Further, it is used most frequently in treating PD according to our previous data mining analysis from literature [48]. In addition, recent RCTs have shown that SP6 stimulation could relieve the abdominal pain and improve the menstrual pain-related symptoms [49, 50]. Thus, SP6 is claimed as a key acupoint for PD. Conversely, GB39 is an acupoint of gallbladder meridian located at the same level of SP6 on the extremity, and it is usually used to treat migraine, stiff neck back pain, shoulder pain, and so forth, but there are few reports on treatment for gynecologic indications. Thus, SP6 is claimed as a key acupoint for PD and the treatment-irrelevant acupoint GB39 (in the same spinal segments of SP6) is used as control points.

However, no significant differences were found in EA at SP6 versus above controls for lowing menstrual symptoms assessed by RSS-COX1 [28, 30, 37] and RSS-COX2 [28, 30, 37, 41], which was also supported by the meta-analysis regarding the influence of menstrual pain on daily life assessed by VRS [28, 30, 37]. This finding does not support the positive effects of acupuncture on PD in the majority of previously published studies [51]. A possible explanation for this nonsignificant finding is that RSS-COX1 and RSS-COX2 were used to evaluate the total frequency and average severity of dysmenorrhea symptoms monthly [44], which should be assessed at end of the menstrual period and not immediately after interventions. Furthermore, immediate changes on menstrual pain after interventions assessed by VAS are more sensitive and understandable than VRS [52].

On the other hand, regarding curative rate, the outcome proven by three studies [39, 40, 43] showed that EA stimulation was more effective than conventional drug therapy after a course of treatment (three menstrual cycles or more). Notably, curative rate has not been validated and should be interpreted with caution. However, apart from curative rate, outcome measures of pain relief (VAS) and other menstrual symptoms (RSS-COX1 and RSS-COX2) have not been applied in the three studies to investigate the cumulative and long-term effect of EA on women with PD. Correspondingly, objective and quantitative assessments of PD should be collected by future RCTs to overcome the limitations of previous studies.

Consistent with our current report, some previous meta-analyses of nonpharmacological interventions on PD focused on the acupuncture [27], acupressure [25, 53], moxibustion [54], aromatherapy massage [55], Chinese herbal medicine [56], transcutaneous electrical nerve stimulation [57], vitamin E [58], and oral Ginger [59]. Meanwhile, many meta-analyses about EA have emerged in the recent three years, indicating that EA could provide a positive therapeutic effect for cardiac anesthesia and intensive care [60], knee osteoarthritis [61], acute ischemic stroke [17], and tinnitus [62]. To the best of our knowledge, this is the first comprehensive systematic review and meta-analysis of RCTs on EA in the treatment of PD.

### 4.2. Limitations

The key strength of this study is that all the included RCTs were evaluated as “low” or “moderate” risk of bias in four domains based on the Cochrane collaboration RoB tool. There were also some limitations to consider in interpreting our study. First, our search did not include data in languages other than Chinese and English, which may generate a sampling bias. Further, although 4/9 trials were published in English, the populations involved in the included RCTs were all Chinese. No multicentered study with PD women of different races was gathered and thus EA therapy for non-Chinese populations still remains uncertain. Second, the methodological quality of the included trials was often suboptimal. Randomization, blinding, sample-size calculation, and the handling of all data should be reported specifically, as these are the principal standards of rigorous study design [63]. Although 7/9 studies described the specific methods of random sequence generation, only three studies declared allocation concealment. In addition, none of the included trials reported any details of blinding or the sample-size estimation. Low quality of the included studies may cause overestimation of the treatment effects and thus limit our confidence in the results of this meta-analysis. Third, a certain degree of heterogeneity was observed in some of the meta-analyses in this systematic review. To gain a more in-depth understanding of the overall evidence of EA for PD, RCTs of different treatment schemes, time of application, duration of stimulation, and acupoints selected were included in our systematic review, which may give rise to clinical heterogeneity and thus may negatively affect our results. Finally, some RCTs did not use recognized reliability and validity outcome measurements on PD study, especially the clinically relevant outcomes (e.g., VAS, RSS-COX1, and RSS-COX2). Specifically, this review did not include data on the long-term efficacy of EA in reducing the abdominal pain or improving the menstrual symptoms, since none of the included RCTs evaluated these outcomes after a course of treatment. To judge whether EA is effective for treating PD in future studies, future trials on the evaluation of therapeutic effects should be in compliance with international standards.

### 4.3. Implications for Practice

With the ever-growing interest in complementary and alternative treatments for chronic disease, there has increasingly been attention directed at EA for PD practices. In terms of pain intensity, six studies reported that EA at SP6 acupoint had a significant effect on cramping pain (assessed using a VAS) compared with GB39, nonacupoint, and waiting-list control in the short term. Specific acupoint is defined as points situated in meridian line with the strongest and the most concentrated power for certain disease. According to the principles of Chinese medicine, SP6 is the junction point of spleen, liver, and kidney meridians and is closely related to lower abdomen and uterus. Therefore, SP6 is commonly applied in clinical practice for alleviating dysmenorrhea, presenting preferred instant analgesic effect compared with irrelevant acupoint GB39 on gallbladder meridians and nonacupoints absent from meridian line. Moreover, there was greater prevalence of curative rate in the EA therapy compared with the pharmacological treatments in the long term. Taken together, our findings support that EA at SP6 acupoint should be recommended for patients with PD.

### 4.4. Implications for Research

Considering the above limitations, more well-designed, rigorous, and large RCTs would facilitate an evidence base that can more decisively provide estimates of EA for PD. To improve methodological quality of clinical trials, further RCTs of EA for PD should use the CONSORT statement [60] and revised Standards for Reporting Interventions in Clinical Trials of Acupuncture (STRICTA) [61] as a guideline. Furthermore, clinically relevant outcomes, such as pain intensity and dysmenorrhea-related symptoms, should be addressed and evaluated using validated measurement scales such as a Short-Form McGill Pain Questionnaire or VAS for pain and the RSS-COX1 and RSS-COX2 for related symptoms. Finally, the electrical characteristics of EA, including the electric device, wave length, and frequency, are worthy of further investigation in the future study.

## 5. Conclusions

Our findings indicated that EA at SP6 can provide considerable immediate analgesic effect for PD and its immediate effect of pain relieving seems to be superior to control interventions. Moreover, there was greater prevalence of curative rate in the EA treatment group compared with the pharmacological treatments after a course of treatment. These results appear to be encouraging, but it should be considered at the same time that they are based on relatively low number of trials and relatively poor methodological quality of the primary studies. Hence, future research should be designed strictly and comprehensively to provide unbiased evidence about the efficacy of EA in the treatment of PD.

## Figures and Tables

**Table 1:** Main characteristics of included RCTs.

Study	Sample size	EA	Control	Duration	Outcome
Liu et al., 2011	E: 49 C1: 49 C2: 48 C3: 48	at SP6, 2/100 Hz, 30 min 0.5–1.6 mA, HANS-200	C1: at GB39, 2/100 Hz, 30 min C2: nonacupoints, 2/100 Hz, 30 min C3: waiting-list	Once/day, for 3 days	VAS, VRS, RSS

Liu et al., 2014	E: 167 C1: 167 C2: 167	at SP6, 2/100 Hz, 30 min 0.5–1.6 mA, HANS-200	C1: at GB39, 2/100 Hz, 30 min C2: nonacupoints, 2/100 Hz, 30 min	Once/day, for 3 days	VAS, VRS, RSS

Liu, 2016	E: 50 C: 50	at ST34 and ST36, 30 min G6805-2	C: Tianqi Tongjing Capsule	5 days/MC, for 3 MCs	Curative rate

Ma et al., 2010	E1: 13 C1: 14 C2: 12 C3: 13	at SP6, 2/100 Hz, 30 min HANS-200	C1: at GB39, 2/100 Hz, 30 min C2: nonacupoints, 2/100 Hz, 30 min C3: waiting-list	Once/day, for 3 days	VAS, VRS, RSS, uterine arteries hemodynamics

Ren and Zhuang, 2010	E: 30 C: 30	at SP6 and BL32, 2/100 Hz, 30 min 2–5 mA, G6805-B	C: ibuprofen 600 mg/day	5 days/MC, for 3 MCs	Curative rate, uterine arteries hemodynamics

Shi et al., 2011	E1: 10 C1: 10 C2: 10 C3: 10	at SP6, 2/100 Hz, 30 min LH202H	C1: at GB39, 2/100 Hz, 30 min C2: nonacupoints, 2/100 Hz, 30 min C3: waiting-list	1 day	VAS, plasm PG

Song et al., 2015	E1: 163 C1: 165 C2: 164	at SP6, 2/100 Hz, 30 min HANS-200	C1: at GB39, 2/100 Hz, 30 min C2: nonacupoints, 2/100 Hz, 30 min	Once/day, for 3 days	VAS, RSS

Xin et al., 2014	E1: 125 C1: 123 C2: 120	at SP6, 2/100 Hz, 30 min HANS-200	C1: at GB39, 2/100 Hz, 30 min C2: nonacupoints, 2/100 Hz, 30 min	1 day	VAS

Zhi, 2007	E: 57 C: 57	at SP6, 60 Hz, 30 min G6805-2A	Ibuprofen 600 mg/day	5 days/MC, for 3 MCs	Curative rate

E: experimental group; C: control group; MC: menstrual cycle; VAS: visual analogue scale; VRS: verbal rating scale; RSS: retrospective symptom scale.
